# Recent advances in detection techniques for vitamin analysis: A comprehensive review

**DOI:** 10.1016/j.fochx.2025.102226

**Published:** 2025-01-31

**Authors:** Xiangrui Li, Huan Lv, Wencan Luo, WenJia Yang, Linghong Kong, Qiujin Zhu, Lu Zeng

**Affiliations:** a Key laboratory of Plant Resource Conservation and Germplasm Innovation in Mountainous Region (Ministry of Education), School of Liquor and Food Engineering, Guizhou University, Guiyang 550025, Guizhou Province, China; b School of Liquor and Food Engineering, Guizhou University, Guiyang 550025, China

**Keywords:** Water-soluble vitamins, Fat-soluble vitamins, Vitamin analysis, Electrochemical analysis, Immunoassays

## Abstract

Vitamins are vital micronutrients that play critical roles in human growth and development. However, vitamins are highly susceptible to degradation by light, heat, oxygen, and interactions with other food components during processing and storage. Additionally, insufficient intake or malabsorption can lead to vitamin deficiencies, resulting in various diseases. Since the human body cannot synthesize most vitamins, they must be sourced through diet or supplementation. Therefore, vitamin analysis is critical for meeting human nutritional needs and ensuring quality control. In recent years, significant advancements have been made in vitamin analysis. Here, we propose a comprehensive and critical evaluation of detection methods for water- and fat-soluble vitamins that have been studied over the past five years, including microbiology-, spectroscopy-, liquid chromatography–mass spectrometry-, electrochemistry-, sensor-, and immunoassay-based analysis techniques. Notably, immunoassays are highlighted for their simplicity, affordability, and high sensitivity. Finally, the current challenges and prospects of vitamin analysis are discussed.

## Highlights

• The crude extraction and purification methods for water-soluble and fat-soluble vitamins were outlined.
• Advances on optical, chromatographic, electrochemical and immunoassay methods for vitamins were reviewed.
• Hapten design, antibody production, and immunoassays of vitamins were elaborated.
• The challenges and perspectives for the detection of vitamins were also discussed.

## Graphic abstract

Detection of vitamins by microbiological assay, spectroscopy, chromatography, electrochemical methods, sensors, and others.

**Figure:**
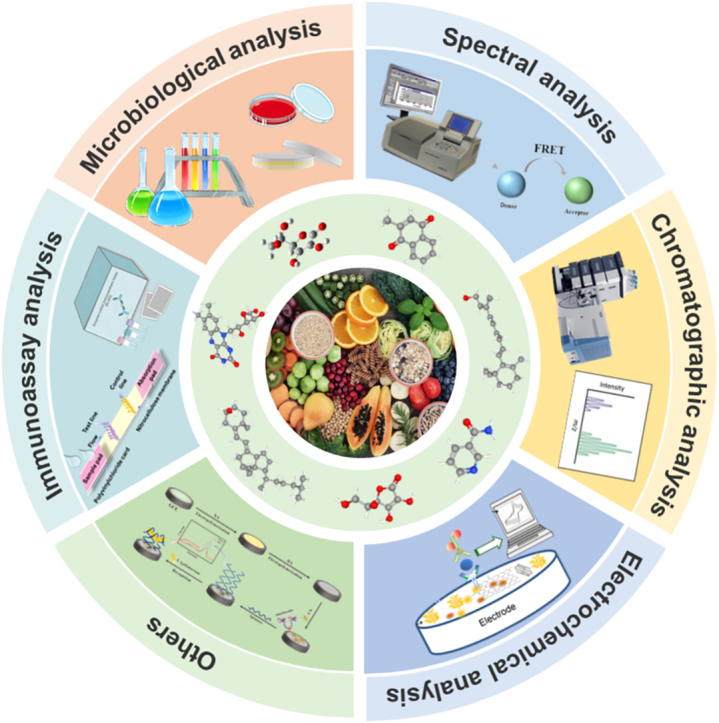

## 1 Introduction

Vitamins, a diverse group of structurally heterogeneous organic micronutrients, are essential for metabolic processes involving carbohydrates, lipids, and proteins, and they play a fundamental role in human development, growth, and self-sustainment (Cara et al., 2024; Lampousi et al., 2024). Since the human body cannot synthesize most vitamins, they must be sourced daily from their diet. However, vitamins are chemically unstable and easily degraded by heat, light, oxygen, and interactions with other food components. Furthermore, poor dietary habits, insufficient intake, malabsorption, and certain diseases can lead to vitamin deficiencies. Vitamin deficiency is a significant global health issue affecting over two billion people (Hicks et al., 2019). Vitamin deficiencies are linked to various adverse health outcomes, including impaired physical and mental development in children, increased susceptibility to disease, intellectual disabilities, blindness, reduced productivity, and even death in severe cases (Gombart et al., 2020). For example, vitamin B_12_ (VB_12_) deficiency causes anemia, affecting an estimated 42 % of children worldwide, with approximately 20 % of maternal deaths being linked to this condition (Morotti et al., 2023). Vitamin A (VA) deficiency is associated with visual impairment or blindness, impacting approximately 29 % of the population in low- and middle-income countries (Buzigi et al., 2022). Moreover, approximately one billion people have insufficient levels of vitamin D (VD), and VD deficiency, which leads to rickets, has been reported across all races and age groups (Dey et al., 2024).

The aforementioned issues have yielded significant interest in nutritional supplements and vitamin-fortified foods. Vitamins are added to food as additives to compensate for the loss of vitamins during cooking, food processing, and long-term storage (R. Kumar, Singh, et al., 2023). Additionally, industrial vitamin products, such as complex vitamin supplements, can reduce the risk of deficiency. Many food and pharmaceutical companies are researching the production of functional foods, including vitamin-fortified foods, such as fortified grains, milk, and water. Additionally, functional beverages rich in dietary multivitamins and multiple mineral supplements are very popular in modern society. Consequently, the U.S. Food and Drug Administration (FDA) has mandated nutritional labeling for food and beverages, which serves as a benchmark for evaluating daily vitamin intake (McBurney et al., 2017). Similarly, the European Parliament and Council's Regulation (EC) No. 1925/2006 requires the inclusion of both endogenous and exogenous vitamins and minerals on food labels (Turck et al., 2021). The National Standard for Food Safety (GB 13432–2013) stipulates the nutrients that must be labeled. Therefore, the development of sensitive, accurate, fast, and reliable vitamin analysis methods is essential for minimizing vitamin loss during production, meeting human health needs, and increasing the nutritional value of food. Moreover, these methods ensure timely and accurate data evaluation during quality supervision, thereby protecting consumer health.

Recently, there has been a significant increase in the number of reports on vitamin detection methods. With advancements in analytical technology, vitamin analysis has evolved from the detection of single analytes to the simultaneous detection of multiple vitamins. Additionally, the sensitivities of new analytical methods continue to improve, and the detection times have been notably reduced. These detection methods mainly include spectroscopy-, liquid chromatography–mass spectrometry (LC–MS)-, electrochemistry-, sensor-, and immunoassay-based analyses. Among these methods, LC–MS is the most frequently reported method for detecting vitamins, followed by spectroscopy-, electrochemistry-, sensor-, and immunoassay-based analysis methods. Analytical methods for detecting vitamins have advanced quickly, with improvement initiatives being focused on making these methods more rapid, cost-effective, and efficient. However, traditional methods, such as high-performance liquid chromatography (HPLC) and LC–MS, are often expensive, time-consuming, and labor-intensive, limiting their applicability in rapid detection. The development of nanomaterials has innovated spectral, electrochemical, and biosensor analysis techniques. However, specific issues, such as stability, accuracy, and reproducibility, continue to challenge their practical application. Immunoassays, which are based on specific antigen–antibody interactions, are a promising alternative for high-throughput vitamin analysis, but they require ongoing refinement to increase their effectiveness.

In this work, we summarized the existing pretreatment methods for vitamin analysis and reviewed the relevant detection methods from the past five years. We primarily focused on the progress made by immunoassays in vitamin detection. Finally, we discussed the prospects of vitamin analysis, offering important references for further development in the field.

## 2 Sample pretreatment

Several factors influence the performance of vitamin analysis, including the stability of the analyte, such as heat, pH, light, and oxygen sensitivity; the low concentrations of target analytes; the variety of food types; the complexity of the food matrix; interactions between analytes and other components, such as proteins, lipids, and sugars; and the presence of multiple active forms of the analyte. Sample pretreatment is a critical step in analysis; purification before analysis can reduce signal interference, minimize instrument contamination, and mitigate matrix effects in complex samples (Gonzalez-Martin et al., 2023). Moreover, this step significantly enhances the sensitivity, accuracy, and reproducibility of vitamin analysis.

### 2.1 Crude extraction

Water-soluble vitamins (WSVs) are chemically unstable and highly susceptible to degradation by light, pH, oxygen, heat, metals, and enzymes. Therefore, all prepared samples should be protected from light exposure. Sample preparation involves acid hydrolysis, enzymatic treatment, or protein precipitation to release bound vitamins from the food matrix. Common acids used for hydrolysis include hydrochloric acid (HCl), perchloric acid (HClO_4_), sulfuric acid (H_2_SO_4_), and metaphosphoric acid (MPA). Specific proteases, such as papain, acid phosphatase, pepsin, and amylase, are employed for protein hydrolysis. Protein precipitation methods, such as salting out, organic solvent precipitation, isoelectric point precipitation, heat treatment, and enzymatic hydrolysis, are commonly used to extract WSVs from protein-rich samples. In a recent study, vitamin B_2_ (VB_2_) was successfully extracted from milk using HCl, and then enzymatic hydrolysis was performed (Laverroux et al., 2021). VB_12_ was released by the precipitation of milk proteins with ethanol and was detected via dual HPLC systems (Bodur et al., 2021). In another study, scholars (Shetty et al., 2020) used cold methanol to precipitate milk proteins and ether to remove cholesterol, and they analyzed the B vitamins in the sample using ultrahigh-performance liquid chromatography–mass spectrometry (UPLC–MS).

Fat-soluble vitamins (FSVs) are a series of homologs that are present in low concentrations in food matrices, and they interact with other food components, such as polysaccharides, proteins, and lipids. FSVs are sensitive to light, heat, oxygen, acids, and bases, chemically heterogeneous, and susceptible to high-temperature isomerization. To release FSVs effectively from protein or lipid matrices, methods such as protein precipitation, saponification, and enzymatic hydrolysis are employed. Among these techniques, saponification is commonly employed to extract fat-soluble vitamins (FSVs) from lipid-rich samples. During saponification, a 50 % (*w*/*v*) aqueous solution of KOH or NaOH is typically combined with ethanol as a solvent. Following this reaction, hexane and other organic solvents that are immiscible with water are used to extract the vitamins. However, this method is unsuitable for extracting vitamin K (VK) and its homologs, which decompose easily under alkaline conditions at high temperatures. Cold saponification is an alternative suitable for extracting FSVs that are prone to decomposition and isomerization at elevated temperatures. Saponification combined with solid–liquid extraction (SLE) and liquid–liquid extraction (LLE) remains the most effective approach for extracting FSVs, and it has been applied to various substrates, including milk, milk formulas, rice, cereals, bread, and baby food (Katsa et al., 2021).

### 2.2 Clean-up methods

The cleaning of a crude sample primarily reduces the concentrations of proteins, lipids, and other substances, but it does not effectively separate vitamins. For example, vitamins are typically bound to proteins, lipids, and phosphate groups. Crude extraction primarily reduces the concentrations of these substances without effectively isolating the vitamins, which remain in an aqueous phase. The highly polar nature of vitamins makes it challenging to enrich and elute them from water using conventional solvent extraction or sorbents, necessitating further purification and concentration.

Subsequent purification techniques include solid-phase extraction (SPE) (Arvapally et al., 2021), SLE, solid-phase microextraction (SPME) (Qi et al., 2021), supercritical fluid extraction (SFE) (Oberson et al., 2020), LLE (Arachchige et al., 2021), and liquid–liquid microextraction (LLME) (Aresta et al., 2020). Various adsorbents, such as metal–organic frameworks (MOFs), supramolecular polymers (SPs), molecularly imprinted polymers (MIPs), and affinity adsorbents, have been developed and integrated with sample pretreatment methods, such as magnetic solid–phase extraction (MSPE) and dispersive solid–phase extraction (DSPE), to further enrich target compounds from complex matrices (Lhotska et al., 2024).

For example, scholars (Perez-Cejuela et al., 2020) prepared a biological metal–organic framework (bio-MOF) based on a natural L–amino acid-disubstituted oxamide ligand as an adsorbent for the recognition and extraction of B vitamins from fruit juices and energy drinks. Compared with a commercial C18 cartridge, the bio-MOF, which was evaluated as an SPE sorbent, demonstrated good effectiveness. Additionally, scholars (Y.-Z. Ding et al., 2022) designed and synthesized a novel spinel particle ZnFe_2_O_4_@PANI that was characterized by a high specific surface area, efficient magnetic separation process, selective adsorption capacity, and resistance to matrix interference. Vitamin B_9_ (VB_9_) was quantitatively detected in rice samples taken from different years by ultrahigh-performance liquid chromatography/tandem mass spectrometry (UPLC–MS/MS). Furthermore, VB_9_-fortified rice and wheat flour were extracted and purified by enzymatic hydrolysis and immunoaffinity columns and were detected via HPLC (Mahato et al., 2020). Researchers have developed a novel polymer material, PAA-T, which can be used as a solid adsorbent for the simultaneous determination of vitamins A and D in food samples via solid-phase extraction (Koseoglu et al., 2020). Scholars (Bagheri et al., 2023) synthesized magnetic layered double hydroxide/MOF composites (IRMOF–3@MLDH) with abundant adsorption sites due to their porous structures and high surface areas. This characteristic makes them effective SPE adsorbents that are particularly suitable for retaining vitamin D_3_ (VD_3_). The linear range of microfluidic solid-phase extraction using the adsorbent is 5–2000 ng/mL, with a limit of detection (LOD) of 1.4 ng/mL. Compared with traditional silicon-based adsorbents, IRMOF-3@MLDH composites exhibit greater sensitivities for the determination of VD_3_.

### 2.3 Eco-friendly sample preparation methods

**Fig. 1:**
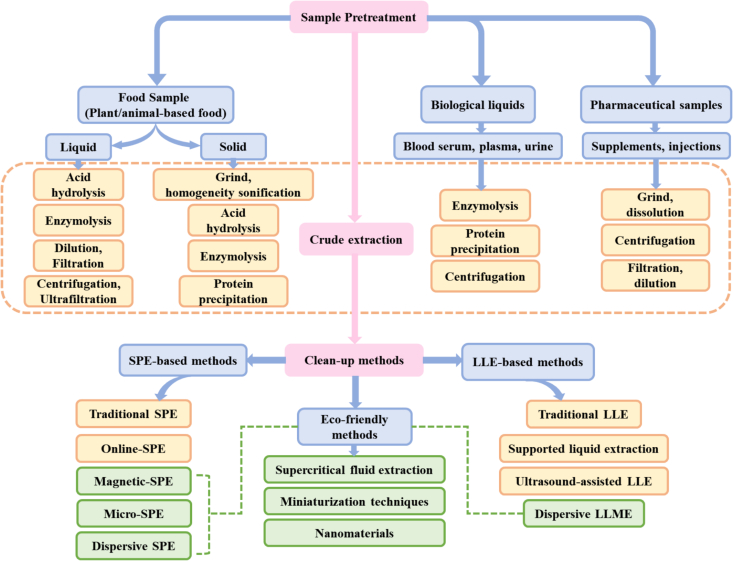
Sample pretreatment scheme for vitamins in different matrices.

Traditional sample pretreatment techniques often rely on large quantities of toxic organic solvents, which are difficult to automate, time-consuming, and labor-intensive, resulting in reduced extraction efficiency. Recent advancements in analytical chemistry emphasize the development of environmentally sustainable and green extraction methods to reduce the consumption of hazardous chemicals and their impacts on the environment. Miniaturization techniques that reduce costs, consume toxic and harmful organic solvents, and require low sample volumes are increasingly being adopted. For example, scholars (Xie et al., 2021) employed deep eutectic solvent-based liquid phase microextraction (DES–LMPE) to extract four tocopherol isomers from edible oils, achieving optimal efficiency with a 1:2 tetrabutylammonium chloride-to-ethanol molar ratio of. Other scholars (Aresta et al., 2020) compared three sample pretreatment methods, namely, SLE–LLE, SLE, and SPME, and reported that all methods were suitable for analyzing onion, carrot, celery, and kale samples. However, SPME performed the best while reducing solvent use, time consumption, and experimental complexity. Some scholars (Dal Bosco et al., 2021) developed a hydrophobic eutectic solvent composed of L-menthol and butylated hydroxytoluene, which was applied for the first time in dispersive LLME (DLLME) to separate carotenoids and FSVs from fruit juices. Nanomaterial-based extraction techniques have great potential for the pretreatment of food matrices. The unique properties of nanomaterials allow for the miniaturization of extraction and purification procedures, minimizing solvent and sample usage. Researchers (Memarbashi Avval & Khani, 2022) employed the green supramolecular solvent-based liquid–liquid microextraction (Ss–LLME) method to preconcentrate and quantify riboflavin via fluorescence. This supramolecular solvent (Ss) interacts with the analyte through various mechanisms, enhancing extraction efficiency. Additionally, the use of this solvent minimizes the volume of extraction solution needed, significantly reducing the excessive discharge of organic solvents. Scholars (Han, Qiu, et al., 2020) created polystyrene/polypyrrole (PS/PPy) nanofibers via the in situ polymerization of PPy on the surfaces of PS nanofibers through electrospinning. These nanofibers, combined with SPE, were used to isolate vitamin D_2_ (VD_2_) and vitamin D_3_ (VD_3_) from dairy products. Researchers (Koseoglu et al., 2020) synthesized a novel adsorbent tetracycline-grafted polyacrylamide polymer (PAA-T), which was employed in SPE to detect VA and vitamin E (VE). The sample preparation scheme for analyzing vitamins is shown in Fig. 1.

## 3 Current methods for vitamin analysis

### 3.1 Microbiological analysis

Microbiological assay (MBA) is the official method for WSV analysis and is certified by the Association of Official Analytical Chemists (AOAC). This method operates on the principle that microorganism growth is proportional to the vitamin content in the medium (Jamieson et al., 2020). The method involves extracting WSVs from the food matrix through pretreatment, incubating the extracts with a growth medium and microbial culture, and determining the vitamin content using turbidimetry or densitometry, which allows for the indirect quantification of WSVs in the sample.

The development of the VitaFast® system provides a significant improvement upon MBA. This system involves 48- or 96-well microtitration plates coated with specific microorganisms to indirectly determine the WSV content by measuring microbial turbidity. This process eliminates the need to maintain stored cultures, reduces the sample processing time, and allows for high-throughput detection. Currently, the system can determine vitamin B_1_ (VB_1_), VB_2_, vitamin B_3_ (VB_3_), vitamin B_5_ (VB_5_), vitamin B_6_ (VB_6_), vitamin B_7_ (VB_7_), VB_9_, and VB_12_. However, the system requires the manual addition of an extraction solution, which is time-consuming, and the VitaFast® system is relatively costly.

### 3.2 Spectral analysis

Spectral analysis methods can be used to qualitatively identify chemical compositions and quantitatively analyze substance contents on the basis of the characteristic spectra of different compounds. Spectral analysis is widely used for vitamin determination because of its advantages of being fast, simple, easy to perform, and nondestructive to samples. Common techniques include ultraviolet–visible (UV–Vis) absorption spectrometry and fluorescence spectrometry (FLS).

#### 3.2.1 Ultraviolet–visible spectroscopy

UV–Vis spectroscopy, which measures the absorbance and characteristic absorption peaks of analytical substances within the 200–800 nm wavelength range, is commonly used for qualitative, quantitative, structural, and purity assessments of substances (Cao et al., 2024). UV–vis spectroscopy for detecting vitamins is based on two main principles. One principle is the interaction between vitamins and nanomaterials, leading to changes in the surface plasmon resonance absorption peaks of the nanomaterials. Metal nanoparticles, such as gold, silver, and copper, exhibit localized plasmon surface resonance (LSPR), which can significantly enhance optical properties and make outstanding contributions to the field of colorimetric detection (Palani et al., 2023). For example, researchers (Wei et al., 2020) detected vitamin C (VC) via a colorimetric assay of 4-mercaptophenylboronic acid (4-MPBA) and silver nanoparticles (AgNPs). In the presence of VC, AgNP aggregation is triggered by the reversible esterification of 4-MPBA and the cis-1,2-diol moiety of VC, resulting in a color change. The VC concentration-related color changes can be quantified visually or with a UV–Vis spectrometer, completing the detection process within 10 min. The method demonstrates an LOD of 0.70 μM and a linear range of 1–500 μM and can be successfully applied to assess VC levels in orange juice. However, the stability of metal nanoparticles remains a significant concern in colorimetric detection. Different end-sealing agents and stabilizers, such as amino acids, vitamins, acids, and polymers, are used to functionalize nanoparticle surfaces to prevent aggregation and maintain activity over time. For example, scholars (Jayeoye et al., 2021) used sodium ascorbate (SA) as a stabilizer and reducing agent for gold nanoparticles (AuNPs). The SA-AuNPs, which were used as colorimetric probes for detecting VC, presented an absorption peak at 547 nm. The broad absorption spectrum indicated the incomplete reduction of Au^3+^ to Au°. Through a seed-mediated growth mechanism, VC acted as a reducing agent, fully reducing the amount of Au in the seed solution and forming AuNPs that were deposited on the SA–AuNP seeds. This process caused a blueshift in the absorption peak and increased the absorption intensity. The method showed a linear range of 12.5–150.0 μM, with an LOD of 11.2 μM, and was further applied to detect VC in VC injections and fruit juice.

Another principle is based on the catalytic properties of oxidase- or peroxidase-like materials or peroxidase itself, which promote substrate color development, where the addition of vitamins causes changes in absorbance, thereby enabling vitamin detection. Various materials, including magnetic nanomaterials (MNMs), nanosheets, MOFs, covalent organic frameworks (COFs), metal-based nanozymes, atomic catalysts (ACs), and nanocomposites, are increasingly used for vitamin detection, particularly for VC. For example, researchers (X. Wang, Chen, & Zhao, 2023) detected VC using the oxidase activity of Fe₃O₄@SiO₂@NiCo₂S₄ bimetallic magnetic nanocomposites. In this reaction system, dissolved oxygen adsorbed on the Fe₃O₄@SiO₂@NiCo₂S₄ surface undergoes electron transfer, generating reactive oxygen species (ROS), such as O_2_·^−^, ^1^O_2_, and H_2_O_2_. H₂O₂ is further decomposed into the hydroxyl radical •OH, which oxidizes 3,3′,5,5′-tetramethylbenzidine (TMB) to produce a blue-colored product (ox-TMB). After a certain amount of ascorbic acid (AA) is added, the color of the solution changes from blue to colorless.

#### 3.2.2 Fluorescence spectrometry

FLS is primarily used to detect target analytes through fluorescence quenching (fluorescence turn-off) or fluorescence enhancement (fluorescence turn-on) (Mal et al., 2024). The quenching mechanisms include the inner filter effect (IFE), static quenching, photoinduced electron transfer (PET), dynamic quenching, and fluorescence resonance energy transfer (FRET) (Marimuthu et al., 2025). In static quenching, a nonfluorescent ground-state complex forms between the fluorophore and quencher without altering the fluorescence lifetime. In contrast, dynamic quenching arises from collisions between excited fluorescent molecules and the quencher, changing the fluorescence lifetime. In addition, both FRET and IFE involve energy transfer processes: FRET involves the nonradiative transfer of energy from an excited donor fluorophore to a proximal acceptor molecule, which relies on spectral overlap, proper dipole orientation, and a donor–acceptor distance of less than 10 nm (Li, Wang, et al., 2024). FRET is linked to dynamic quenching, which impacts the fluorescence lifetime. Table 1 summarizes the FRET-based analytical methods for vitamin detection. For example, scholars (Li, Wang, et al., 2024) synthesized blue fluorescent copper nanoparticles (F-CuNPs) with a high fluorescence quantum yield. A significant spectral overlap was observed between the emission spectra of F-CuNPs and the UV–Vis absorption spectra of VB_2_. The fluorescence lifetimes of F-CuNPs were reduced after the addition of VB_2_, indicating the occurrence of FRET between F-CuNPs (donors) and VB_2_ molecules (acceptors). The FRET-based system enabled the detection of VB_2_ with a linear detection range of 0.51 nM–34.64 nM and an LOD of 0.25 nM.

**Table 1:** FRET-based analytical methods for determination of vitamins.

Analyte	Donor	Acceptor	Linear range	LOD	Real samples	Ref
VB_2_	F-CuNPs	VB_2_	0.51–0.346 nM	0.25 nM	VB_2_ tablet, orange	(Wang, Mu, et al., 2023)
VB_2_	CDs	VB_2_	0.1–3.0 μg/mL	1.0 ng/mL	Multivitamin/mineral supplements	(Monte-Filho et al., 2019)
VB_2_	NPCDs	VB_2_	0.5–50 μmol/L	0.17 μmol/L	Milk, riboflavin pharmaceutical tablets	(Lin et al., 2019)
VB_2_	Eu-BPTA, Sm-BPTA, Dy-BPTA	VB_2_	ND	5.02 nM, 8.56 nM and 8.62 nM	Milk, fetal bovine serum, energy drinks	(X.-C. Sun et al., 2022)
VB_2_	Mussels-Derived Carbon Dots	VB_2_	1–10 μmol/L	6.06 nmol/L	Milk, milk powder, riboflavin pharmaceutical tablets	(Zhao et al., 2022)
VB_2_	CD	VB_2_	0-11 μM	0.025 μM	Energy drink, green tea, wine	(Sotolongo-Garcia et al., 2021)
VB_2_	CNDs	VB_2_	0.35-35.9 μM	37.2 nM	Orange beverage, soybean milk, milk, honey, multivitamin tablet, VB complex tablet	(Du et al., 2021)
VB_2_	g-CNQDs@Zn-MOF	VB_2_	0.005–1.0 μM	15 nM	Milk, VB_2_ tablet	(Feng et al., 2021)
VB_2_	Up-conversion fluorescence nanofibers embedded with Ag@SiO2	VB_2_		0.1 μg/mL	Human body fluid	(J. Zhang et al., 2020)
VB_9_	N, S, I-CDs	VB_9_	0.1–175 μM	84 nM	Living cells	(Mu et al., 2020)
VB_12_	N-doped CDs	VB_12_	0–100 μM	2.19 μM	Human blood serum	(Yu et al., 2019)
VB_12_	Mn^2+^-doped ZnS QDs	VB_12_	4.9–29.4 pM	1.15 ± 0.06 pM	ND	(Pramanik et al., 2020)
VB_12_	Boron-doped CDs	VB_12_	0.20–30 μM	8.0 nM	Mineral water, vitamin drink, VB_12_ tablets	(Jia et al., 2019)
VC	PUC3 and PUC4 Zn-based MOFs	AA	ND	0.032 μM and 0.025 μM	Aqueous	(A. Kumar, Mutreja, et al., 2023)
VC	CdS QDs	PDA	5.0–100.0 μmol/L	1.16 μmol/L	VC tablets	(P. Li et al., 2022)
VC	CoOOH nanoflakes	CQD	0.1–30 μM	0.031 μM	Fresh orange	(Peng et al., 2022)
VC	MnO_2_ nanosheets	AuNCs	1.5–100 μmol/L	0.45 μmol/L	Beverage samples	(J. Zhang et al., 2021)
VC	ADD	AA	0.25-190 μM	10 nM	Fruit, vegetables	(Kathiravan et al., 2020)
VC	CD/Ag(I)/AgNP	AA	0–9.0 μM	0.2 μM	Human serum samples	(J. Liu et al., 2019)
VC	MQDs	P_EP_-_PEI_	0.5–40 μM	0.2 μM	Human urine samples	(F. Zhang et al., 2019)
VC	SiMoW	Eu	0.1-0.9 mmol/L	0.53 μmol/L	Human urine and spinach	(Fu et al., 2019)
VC	polyallylamine-AuNCs	MnO_2_ nanosheets	0.01–200 μM	3.2 nM	Biological samples	(Tan et al., 2019)
VA	ZnS QDs	VA	3.33–36.66 μM	1.062 μM	ND	(Safari et al., 2020)
25-OH VD_3_	GO	FAM-Aptamer	0–1.25 μg/mL	0.075 μg/mL	Serum	(Gupta et al., 2021)

IFE, another nonradiative energy transfer mechanism, involves the absorption of both excitation and emission light by a quencher (Weitner et al., 2022). This technique enables the conversion of the absorption signal of the quencher into a fluorescence signal when its absorption band overlaps the excitation or emission spectra of the fluorophore. The detection of vitamins using IFE primarily employs the following principles: (1) vitamin analysis using fluorescence turn-off strategies, where fluorescent materials are quenched by vitamins; (2) fluorescence quenching by an added quencher, in which the addition of a vitamin decomposes the quencher, thereby recovering the fluorescence of the system through a turn-off strategy for vitamin detection; and (3) by combining the two aforementioned concepts, a ratiometric fluorescence system based on dual fluorescence emission is employed for vitamin detection. PET involves the excitation of oxidants or reducing agents to excited states, which is followed by electron transfer to ground state molecules, resulting in the formation of radical ion pairs or charge transfer complexes (Schubert et al., 2021). As a redox process, PET occurs when a molecule in an excited state donates or captures electrons from fluorophores, leading to fluorescence quenching.

In addition to detecting vitamins through the individual fluorescence quenching mechanisms of FRET, IFE, and PET, scholars have recently reported their synergistic application. Researchers (Yang et al., 2021) successfully prepared FL@UiO-67 NCs, which exhibited distinct emission peaks at 398 nm and 546 nm. VC quenched the fluorescence of the FL@UiO-67 NCs, enabling their detection. The mechanism of fluorescence determination for VC using FL@UiO-67 NCs was further investigated. The UV–Vis spectra of VC overlapped the fluorescence excitation spectra of the FL@UiO-67 NCs, indicating the presence of an IFE. FL acted as an electron donor, whereas 4,4′-biphenyl dicarboxylic acid (BDCA) served as an electron acceptor, with AA functioning as a photosensitizer. The highest occupied molecular orbital (HOMO) of FL was close to the HOMO of VC, whereas both the HOMO and lowest unoccupied molecular orbital (LUMO) of BDCA were lower than those of VC. This arrangement facilitated the construction of a bridge between the donor and acceptor, thus promoting PET. Consequently, VC dynamically quenched the fluorescence levels of the FL@UiO-67 NCs through both IFE and PET mechanisms.

A fluorescent sensor is a piece of fluorescence analysis equipment that is widely used in biomedicine, environmental monitoring, food safety, and other fields. These sensors are specifically designed to interact with target molecules, generating fluorescence signals for precise detection and analysis (D. Wu et al., 2017). In recent decades, nanotechnology has significantly advanced the development of fluorescent nanomaterials for vitamin detection, including fluorescent organic nanoparticles (FONs), fluorescent gold nanoclusters (F-AuNCs), fluorescent copper nanoclusters (F-CuNCs), fluorescent silicon nanoparticles (F-SiNPs), fluorescent MOFs, CDs, carbon quantum dots (CQDs), GQDs, and UCNPs. Typically, fluorescence nanomaterial-based sensors employ one of three response modes: fluorescence off, fluorescence on, or ratiometric fluorescence. The recognition mechanisms of these sensors include chelation-enhanced fluorescence (CHEF), PET, FRET, intramolecular charge transfer (ICT), and aggregation-induced emission (AIE). For example, scholars (Shen & Fan, 2023) prepared *N*-acetyl-L-cysteine-stabilized copper nanoclusters (NAC-CuNCs). Ce^4+^ strongly oxidizes and reacts with VC to generate Ce^3+^ through a redox reaction. The AIE effect induces the aggregation of NAC–CuNCs in the presence of Ce^3+^, increasing their luminescence. The detection range is from 4 to 60 μM, with a lower LOD of 0.26 μM, successfully enabling the determination of VC in soft drinks. This mechanism is based on S,N-codoped GQDs and a VD_3_-mediated PET mechanism. Yingping Li and colleagues (Y. Li et al., 2019) used thiamine nitrate to create nitrogen–sulfur codoped CQDs (N, S-CQDs) as multifunctional fluorescent probes, which selectively and sensitively detect VB_12_ via FRET, achieving an LOD of 15.6 nmol/L. This method has demonstrated a recovery range of 97.5–104.2 % in detecting VB_12_ in milk and various beverages. In addition, multimodal sensing has garnered significant interest. Scholars (Bai et al., 2025) synthesized phosphorus-doped graphite carbon nitride quantum dots (P-CNQDs) using melamine as the carbon and nitrogen source and ammonium phosphate monobasic as the phosphorus source. P-CNQDs were successfully prepared through chemical oxidation and hydrothermal methods, enabling the multimodal detection of Ag^+^, ciprofloxacin (CIP), and VB_2_ via PET, CHEF, and FRET mechanisms. This multifunctional sensor has been successfully applied to real samples, including river water and VB_2_ tablets (M. Lu et al., 2023).

Owing to the complexity of real samples, fluctuations in the sensing conditions may alter the sensing signal. Thus, a robust fluorescent sensor is highly desirable for vitamin analysis. Most existing fluorescent sensors require fluorescence spectrometers, which are often unaffordable and inaccessible in remote or underdeveloped areas, limiting their broad application. The development of portable fluorescent sensors addresses this limitation and enables point-of-care detection. In particular, smartphone-assisted paper-based nano-sensors provide a low-cost, simple, and convenient option for point-of-care detection.

#### 3.2.3 Fluorescence and colorimetric dual-mode detection

The single-signal readout mode can introduce uncertainties due to variations in operator experience and the experimental environment. In contrast, the dual-signal readout mode addresses the limitations of low accuracy and detection efficiency inherent in single-signal systems (R. Sun et al., 2023). Consequently, an increasing number of researchers are dedicated to developing highly sensitive, rapid, accurate, and stable dual-mode fluorescence and colorimetric detection methods. This approach primarily utilizes the oxidase-like or peroxidase-like activities of materials to catalyze the oxidation of benzidine derivatives, which results in products exhibiting fluorescence excitation or emission peaks, along with characteristic ultraviolet absorption peaks. The fluorescence of the probe is quenched by the oxidation products, but in the presence of vitamins, the oxidation reaction is inhibited, resulting in a restoration of fluorescence (A. Wu et al., 2021). Furthermore, in the presence of vitamins, their oxidation products combine with benzidine derivatives to form additional substances with fluorescence excitation or emission peaks and characteristic ultraviolet absorption peaks. Thus, the fluorescence of the probe is quenched when vitamins are present, whereas in their absence, the fluorescence is restored, corresponding with changes in the ultraviolet absorption of the probe (Wang, Lv, et al., 2022). The fluorescence characteristics of the probes and the ultraviolet absorption properties of the substances in the system enable the proportional detection of vitamins through both fluorescence and colorimetric methods. Researchers (N. Li et al., 2020) synthesized CoOOH nanoflakes with oxidase-like properties, which catalyzed the oxidation of *p*-phenylenediamine (p-PD) to form reddish-brown p-PDox. Upon mixing with fluorescent CDs, the fluorescence was quenched by p-PDox. In the presence of VC, a redox reaction occurred between VC and CoCOOH, causing decomposition and collapse of the CoCOOH nanoflakes, thereby maintaining the fluorescence levels of the CDs. VC determination in fruit juice was achieved using smartphone-assisted UV–Vis absorption spectrophotometry and fluorescence spectrophotometry, with an LOD as low as 0.09 μM. Scholars (X. Liu et al., 2021) prepared polyvinylpyrrolidone-stabilized platinum nanoclusters (PVP–PtNCs), which catalyzed the generation of ROS from dissolved oxygen, further oxidizing OPD to OPDox and resulting in a color change from colorless to yellow. Additionally, in the presence of VC, PVP–PtNCs could oxidize VC to DHA, which subsequently reacted with OPD to form fluorescent compounds. Consequently, the oxidase-like activity of PVP-PtNCs could be employed to establish both colorimetric and fluorometric assays for VC detection, with an LOD of 1.17 μM.

### 3.3 Chromatographic-based methods

#### 3.3.1 Chromatographic analysis

Liquid chromatography (LC) is a widely utilized technique for identifying, quantifying, and separating components within complex mixtures (Studzinska et al., 2024). LC is officially certified by the AOAC as the primary method for determining nutrients in various matrices. This technique operates on the basis of the differential interactions of analytes with the stationary and mobile phases, allowing the target analyte to equilibrate between both phases and achieve separation from interfering substances (Shi, Zuo, et al., 2024). LC encompasses HPLC, reversed-phase HPLC (RP–HPLC), hydrophilic interaction LC (HILIC), and ultrahigh-performance LC (UPLC). LC can be paired with various detection techniques, such as ultraviolet (UV) absorption detection, photodiode array detection (DAD), fluorescence detection (FLD), electrochemical detection (ECD), and mass spectrometry detection (MSD), to enable rapid, efficient, and highly sensitive detection. For example, scholars (Faraji et al., 2020) synthesized a new hydrophobic green eutectic solvent (DES), which was employed in DLLME to extract VB_9_ from fortified flour, which was detected via HPLC. The LOD and limit of quantitation (LOQ) were 1.0 ng/g and 3.0 ng/g, respectively, with recovery rates ranging from 91.6 % to 99.7 %. Similarly, researchers (Islam et al., 2022) utilized HPLC–DAD and HPLC–FLD to update the nutritional profiles of VB_5_, pyridoxine (PN), pyridoxal (PL), and pyridoxamine (PM) in raw and processed seafood. The LODs for VB_5_, PN, PL, and PM were 3 mg/100 g, 0.043 μg/100 g, 0.039 μg/100 g, and 0.069 μg/100 g, respectively, demonstrating high sensitivity and reproducibility. The LC method is accurate, sensitive, and reliable, making it ideal for the precise determination of both single vitamins and multivitamins in various matrices and ensuring robust quality control.

#### 3.3.2 Liquid chromatography–mass spectroscopy analysis

LC–MS offers high sample throughput, selectivity, efficiency, and sensitivity, enabling the quantification of targets through the monitoring of multiple fragment ions. Using vitamin fragment structure information, LC–MS can detect low concentrations of vitamins and facilitate multivitamin analysis, particularly in complex sample matrices. Scholars (Y. Z. Ding et al., 2023) developed a transition metal composite ZnFe₂O₄–NH₂/MXene adsorbent for VB₂ extraction from rice, which was combined with UPLC–MS/MS to determine the VB₂ content. The LOD and LOQ were 0.86 ng/mL and 2.98 ng/mL, respectively. The recovery rates ranged from 81.7 % to 102.5 %, indicating acceptable accuracy. Researchers (Dunlop et al., 2021) developed a sensitive and specific analytical method using LC–triple quadrupole–mass spectrometry (LC–QQQ–MS). In that study, food samples were supplemented with isotope internal standards, saponified, extracted, and derivatized, enabling the accurate quantification of VD_2_, VD_3_, 25-hydroxyvitamin D_2_ (25-OH VD_2_), and 25-OH VD_3_ at low concentrations, with LODs ranging from 0.01 to 0.2 μg/100 g.

LC and LC–MS play crucial roles in accurately identifying and quantifying vitamins in various sample matrices. These techniques provide precision, sensitivity, and versatility by exploiting the differential distribution of vitamins between stationary and mobile phases for separation and by generating ionic fragments for further analysis. LC is highly effective at separating multivitamins with high resolution, while LC–MS is a highly sensitive detection technique. Together, these chromatographic methods enable the determination of vitamin content, ensuring quality, safety, and stability.

### 3.4 Electrochemical analysis

**Fig. 2:**
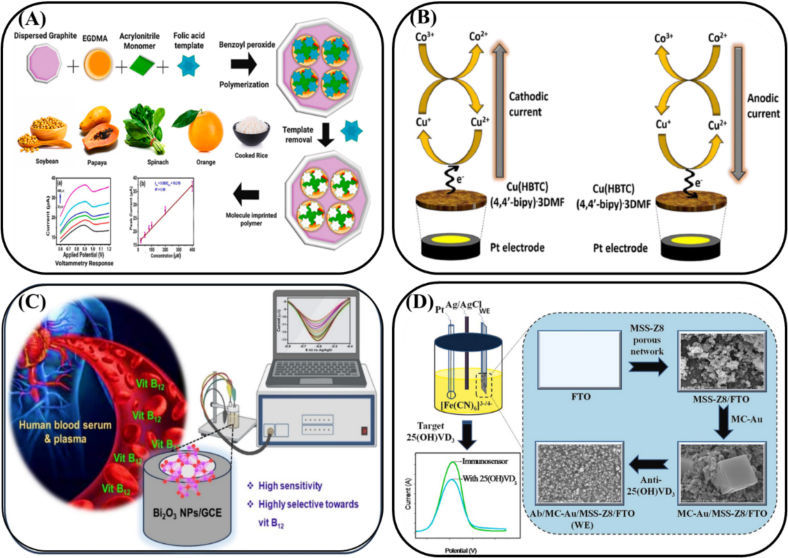
Schematic representation of electrochemical methods for the detection of vitamins. (A) Schematic diagram of fabrication of a molecularly imprinted polyacrylonitrile-imbued graphite-base electrode for the selective detection of VB_9_ in food samples (Bandyopadhyay et al., 2024). (B) Illustration of electrochemical sensing platform based on Cu(HBTC) (4,4′-bipy)·3DMF for rapid detection of VB_12_ in commercial pharmaceutical tablets (Manivel et al., 2019). (C) Scheme of preparation of Bi_2_O_3_ and the application for electrochemical sensing of VB_12_ (Manimaran et al., 2024). (D) Schematic illustration of voltammetric immunosensing MSS-Z8-based platform for the detection of 25-OH VD_3_ (Amandeep Kaur et al., 2023).

Electrochemical analysis is an analytical technique that uses the principles of electrochemistry to measure the composition or concentration of substances on the basis of changes in their electrochemical properties (Sharma, Taneja, et al., 2024). This method is widely employed in vitamin analysis because of its low cost, speed, operational simplicity, high sensitivity, and selectivity. Notably, most vitamins exhibit electrochemical activity, which facilitates their interactions with electrode materials, enabling electron conduction and electrochemical signal generation (Huang et al., 2021). The working electrode is central to electrochemical analysis, as it serves as the site for redox reactions, allowing electron exchange between the electrode and analytes. The selection of electrode material is critical, influencing the selectivity, sensitivity, and stability of vitamin measurements (Y. Wang, Zhang, et al., 2022). Upon application of an electric potential, redox reactions at the electrode surface produce a current, with the process efficiency depending on specific factors, such as electrode conductivity, chemical stability, and surface area. Common electrode materials include metals, carbon-based electrodes, graphite, and conductive polymers (Patil et al., 2025). Metal electrodes, such as gold, are well regarded for their excellent electrical conductivity and corrosion resistance in vitamin testing (Bian et al., 2024). Owing to their high chemical stability and conductivity, platinum electrodes are widely used in various vitamin assays (Shi, Zuo, et al., 2024). Glassy carbon electrodes (GCEs) are notable for their wide potential window, low background current, and strong stability, making them effective for detecting vitamins in complex food matrices (Yenealem et al., 2024). Owing to their high electrical conductivity, mechanical strength, low cost, and large surface area, graphite electrodes are ideal for high-resolution, sensitive vitamin detection (Fig. 2A) (Bandyopadhyay et al., 2024). Conductive polymers, such as PPy and polyaniline (PANI), are of interest because of their superior electrical conductivity and environmental adaptability. Selecting the optimal electrode material is essential for enhancing the accuracy, sensitivity, and reproducibility of electrochemical methods for vitamin assays, with key considerations being conductivity, chemical stability, and surface area (Patil et al., 2025).

#### 3.4.1 Cyclic voltammetry

Cyclic voltammetry (CV) is one of the most widely used electrochemical measurement techniques (Alam et al., 2022). CV typically employs a three-electrode setup consisting of a reference electrode, a working electrode, and an auxiliary electrode. These electrodes form two circuits: the working and auxiliary electrodes form the polarization current loop, whereas the working and reference electrodes form the potential measurement and control loop (Rajendrachari et al., 2022). In the latter circuit, virtually no current is flowing, ensuring that the potential measurement is unaffected by the current in the polarization loop. This configuration allows the current to pass through the working electrode while maintaining the stability of the potential of the reference electrode, thereby enabling the precise measurement and control of both current and potential. Consequently, the relationship between the current and potential at the working electrode can be accurately obtained (B. Li, Dai, et al., 2024).

In a study (Manivel et al., 2019), a novel electrochemical VB_12_ biosensor based on Cu(HBTC)(4,4′-bipy)·3DMF nanorods was developed for the sensitive and selective detection of VB_12_ at low concentrations in real samples. CV analysis revealed that the modified electrode exhibited excellent electrocatalytic redox reversibility for the Co^3+^/Co^2+^ redox couple at −0.192 and − 0.268 V. The sensor demonstrated a sensitivity of 0.104 μA/μM, a low detection limit of 50 nM, and a wide linear range of 0.1–188.2 μM (Fig. 2B). In another study (Zhang et al., 2018), CV was utilized to determine the concentration of VB_2_. Under optimized conditions, the prepared imprinted sensor displayed a good linear response to VB_2_ in wide concentration ranges from 0.002 to 0.9 μM with a low detection limit of 0.7 nM, and successfully applied to electrochemically detect VB_2_ in drug samples with good reproducibility, repeatability and storage stability. Researchers (Gopi et al., 2025) synthesized a nickel–iron bimetallic organic framework (C_x_Ni_x_O_y_Fe_y_O_x+3_), modified it onto a screen-printed carbon electrode for electrochemical analysis, and detected VB_12_ using CV. The results demonstrated excellent reproducibility and stability, with a detection limit for VB_12_ as low as 49 nM.

#### 3.4.2 Differential pulse voltammetry

Differential pulse voltammetry (DPV) is a pulsed voltammetry technique that enables chemical analysis by applying a sequence of small, fixed-amplitude pulses over a linearly varying potential. DPV measures the current difference before and after each pulse, effectively minimizing background signal interference and enhancing sensitivity, which makes it particularly suitable for detecting trace levels of analytes. Scholars (Manimaran et al., 2024) utilized synthetic bismuth oxide nanoparticles to quantify VB_12_ using DPV, achieving a broad linear range from 10 nM to 275 nM, with an LOD of 3.66 nM (Fig. 2C). Similarly, researchers (Bindu et al., 2024) developed an electrochemical detection method for VB_2_ using an aniline-modified TiO_2_ composite carbon paste electrode (AN–MTiO_2_CCPE) prepared via electrochemical polymerization. The scholars examined the electrochemical behaviors of VB_2_ under various parameters and measured VB_2_ concentrations in the range of 0.2 μM to 3.4 μM, indicating an adsorption-controlled reaction. The LOD and LOQ for the redox reaction were 0.28 μM and 0.45 μM, respectively. This approach was successfully applied to the analysis of VB_2_ tablet samples. Scholars (Khalife et al., 2024) developed an electrochemical biosensor utilizing *Lactococcus lactis* NADPH-dependent quinone reductase, employing CV and DPV for rapid, redox-free detection of vitamin K_3_. The LODs in buffer and milk were 0.87 μM and 4.1 μM, respectively. Similarly, researchers (Hashem & Magar, 2024) grafted pectic acid onto poly(acrylamide-coacrylic acid) nanoporous membranes and modified a screen-printed electrode to quantify the VB_2_ concentrations in real samples. This approach, which uses CV and DPV, achieved LODs of 0.004 nM and 0.97 nM, with linear ranges of 0.01–2 nM and 2–90 nM, respectively.

#### 3.4.3 Square-wave voltammetry

Square-wave voltammetry (SWV), a specialized form of DPV, employs a pair of pulses of equal amplitude and opposite direction instead of a linearly increasing step as the signal. Compared with DPV, SWV not only effectively suppresses the background current but also provides a higher current response and scan rate. The advantages of SWV include greater sensitivity, lower detection limits, and a wider dynamic range than DPV, making it particularly suitable for identifying and quantifying trace amounts in complex matrices.

In a study (Martins et al., 2023), SWV was used to monitor multivitamins by modifying GCEs with nitrogen- and sulfur-doped graphene QDs immobilized in chitosan. The SWV parameters were optimized to maximize the current response, and the electrochemical oxidation of vitamins was investigated. Calibration curves for VB_2_, VB_6_, and VB_12_ were constructed with LODs of 0.30, 30.1, and 0.32 nmol/L, respectively. This method successfully quantified vitamins in fruit-based energy drink samples. Additionally, scholars (Thangphatthanarungruang et al., 2020) developed graphene nanocomposites for the simultaneous detection of FSVs (A, D, E, and K) in various matrices. Adsorption SWV was applied to determine the concentrations of vitamins A, D, E, and K in samples of infant milk, yogurt, and parsley. Under optimal conditions, the anodic current exhibited a strong linear relationship with the vitamin concentration in the range of 0.02 to 1 μg/mL. The LODs for vitamins A, D, E, and K were 0.0086, 0.0063, 0.0075, and 0.0071 μg/mL, respectively. Researchers reported the fabrication of a novel electrochemical sensor for the rapid and accurate determination of vitamin B_7_ (biotin) using a shockwave-treated hydroxyapatite/graphene oxide (SW–HAP/GO) composite-modified glassy carbon electrode. SWV measurements of the SW–HAP/GO-modified glassy carbon electrode demonstrated efficient and selective detection of biotin in 0.1 M PBS, with a wide dynamic range of 16.7–611 μM. The lowest detection limit of the sensor was estimated to be 0.215 μM on the basis of the calibration curve (Meenakshi et al., 2024).

In summary, CV, DPV, and SWV are various approaches for detecting vitamins, whereas DPV enhances sensitivity for precise quantification. Owing to its superior sensitivity and broad dynamic range, SWV is particularly well suited for identifying and quantifying vitamins in complex matrices.

### 3.5 Immunoassay analysis

**Fig. 3:**
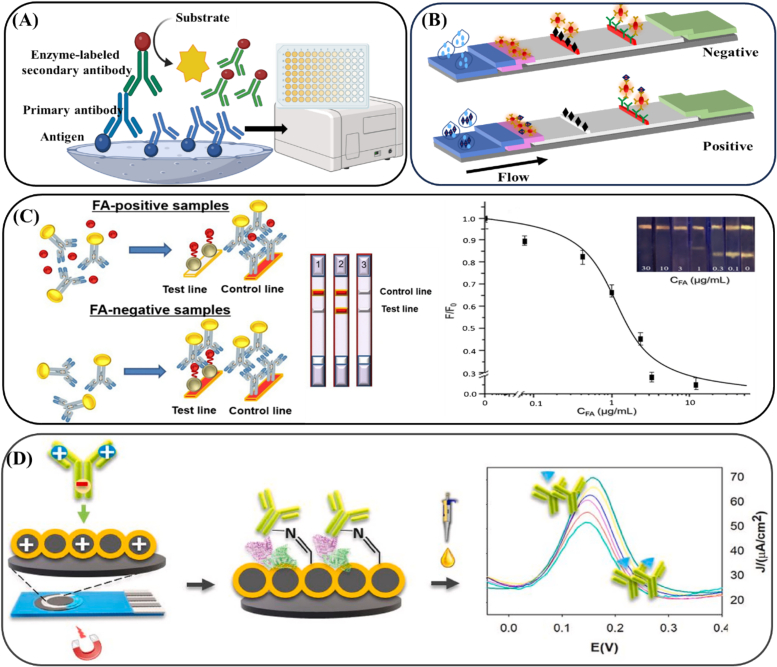
(A) Principle of indirect competitive ELISA. (B) Schematic of the immunochromatographic strip for vitamin analysis. (C) Schematic illustration of determination of folic acid by lateral flow immunoassays based on AgInS/ZnS fluorescent quantum dots (Novikova et al., 2020). (D) Schematic illustration of ASu@MNPs-based electrochemical immunosensor for vitamin D_3_ serum samples analysis (Polli et al., 2023).

Immunoassays have emerged as promising alternative methods for vitamin determination, offering certain advantages, such as simplicity, low cost, speed, and high accuracy. Owing to the highly specific interactions between antigens and antibodies, immunoassays enable the qualitative and quantitative detection of target molecules (S. Liu et al., 2024). Various immunoassay techniques include enzyme-linked immunosorbent assay (ELISA), lateral flow immunoassay (LFIA), fluorescence polarization immunoassay (FPIA), and chemiluminescence immunoassay (CLIA). The immunoassay principles for the analysis of small-molecule compounds are detailed in Fig. 3. This section is focused on the key aspects of immunoassay development, such as hapten design, antigen synthesis, antibody production, immunoassay format development, and advances in vitamin detection methods in food products.

#### 3.5.1 Hapten design and antigen preparation

Rational hapten design is essential for the development of high-affinity immune reagents and highly sensitive immunoassays (Li, Jiang, et al., 2024). Haptens must be designed to maintain the structural integrity and characteristic groups of the parent molecule while adding linking chains that only slightly affect its electronic and structural properties (Li, Shu, et al., 2024). This approach strikes a balance between chemical method feasibility and minimal target molecule modification. An ideal immune hapten of the analyte must be a near-perfect mimetic of the target molecule; that is, the hydrophobicity, electrostatic, electron configuration, and spatial properties of the hapten are highly similar to those of the target molecule, and the skeleton of the target molecule is more exposed to the immune system to elicit an immune response, thereby producing highly specific antibodies against the target molecule (Q. Lu et al., 2024). To form a complete antigen for animal immunization, the designed haptens are conjugated with carrier proteins, such as human serum albumin (HSA), bovine serum albumin (BSA), rabbit serum albumin (RSA), ovalbumin (OVA), keyhole limpet hemocyanin (KLH), or porcine serum albumin (PSA).

#### 3.5.2 Antibody preparation

Immunoassays rely on the specific binding of antigens and antibodies to detect target substances. To date, the antibodies used for vitamin detection mainly include polyclonal antibodies (pAbs) and monoclonal antibodies (mAbs). pAbs are mixtures of antibodies produced by different B-cell clones in response to the same antigen, enabling them to recognize multiple epitopes on the antigen. Their production does not require the cloning of individual B cells, increasing the process efficiency. These antibodies exhibit broad specificity, allowing them to bind to multiple epitopes of a target antigen, which enhances their versatility in detecting complex or low-abundance targets. However, this broad specificity increases the risk of cross-reactivity with similar antigens, potentially leading to nonspecific binding. Additionally, mAbs are the most commonly used because of their high specificity. mAbs are identical antibodies produced by a single B-cell clone (hybridoma) derived from a single parent B-cell (Peltomaa et al., 2022). A single specific epitope is recognized on the antigen, offering greater specificity than do polyclonal antibodies. Once established, mAbs can be produced in large quantities with consistent quality, minimizing batch-to-batch variability. Although their production process is more complex and time-consuming, involving the creation of hybridomas, mAbs are highly stable in cell culture and have long shelf lives (Das et al., 2024). We summarize the reported production of vitamin haptens, antigens, and antibodies, along with their half-inhibitory concentration (IC_50_) values and cross-reactivity (CR), as shown in Table 2.

**Table 2:** Vitamin hapten, conjugation method, antibody parameters reported.^a^

Vitamins	Hapten structures	Conjugation methods	Antibody types	IC_50_	CR (%)	Ref
VB_1_		active ester	mAb	20.71 ng/mL	_	(Zeng et al., 2020)
VB_2_		carbonyldiimidazole	pAb	6.73 ng/mL	_	(*P.*Wang et al., 2013)
VB_2_		carbonyldiimidazole	mAb	8.18 ng/mL	_	(Zeng et al., 2018)
VB_3_		glutaraldehyde	mAb	603.41 ng/mL	_	(Hu et al., 2024)
VB_5_		active ester	mAb	201.31 ng/mL	_	(Zeng et al., 2021)
VB_6_		active ester	mAb	106.6 ng/mL,250.57 ng/mL,400.11 ng/mL	PL: 42.64; PM: 26.65	(Zeng et al., 2022)
VB_7_		active ester	mAb	2.18 ng/mL	_	(Kong et al., 2016a)
VB_9_		active ester	pAb	15.5 ng/mL	Pteroic acid 145.3; Dihydrofolic acid 35.4; Tetrahydrofolic acid 18.5	(T. Zhang et al., 2012)
VB_9_		active ester	mAb	0.12 ng/mL	Pterine 0.6; Pteroic acid 15; Dihydrofolic acid 5.7; Tetrahydrofolic acid 7.5	(Kong et al., 2016b)
VB_12_		active ester	mAb	0.43 ng/mL	Hydroxocobalamin 68.34; Adenosylcobalamin 70.00; Methylcobalamin 82.35	(Kong et al., 2017)
VB_12_		active ester	pAb	aNM	VB_12_ 34.6; Methylcobalamin 27.6; Hydroxocobalamin 14.6; Adenosylcobalamin 38.3; Analogs of VB_12_ 12.2	(L. S. S. Kumar & Thakur, 2011)
25-OH VD_3_		active ester	pAb	15 nmol/L	NM	(Litvinovskaya et al., 2018)
25-OH VD_3_		NM	NM	NM	NM	(Gregorio et al., 2007)
1α,25 (OH)_2_ VD_3_		NM	NM	NM	NM	(Gregorio et al., 2007)
1α,25 (OH)_2_ VD_3_		active ester	pAb	NM	20-epi-1α,25(OH)_2_ VD_3_ 8; 25-OH VD_3_ 1.3；1-hydroxy VD_3_ 1.3; VD_3_ < 0.01	(Blæhr et al., 2003)
1α,25 (OH)_2_ VD_3_		active ester	pAb	NM	20-epi-1α,25(OH)_2_ VD_3_ 50; 25-OH VD_3_ < 0.01；1-hydroxy VD_3_ 1.5; VD_3_ < 0.01	(Blæhr et al., 2003)

a NM represent not mentioned.

#### 3.5.3 Enzyme-linked immunosorbent assay

ELISA is a well-established immunoassay that combines the specificity of antigen–antibody interactions with the catalytic amplification of enzymes and is widely used in food safety, environmental monitoring, and disease diagnosis (Jin et al., 2023). In an ELISA, an antigen is immobilized on microwells and interacts with a specific antibody, which is then detected using enzyme-labeled secondary antibodies. Common enzymes include alkaline phosphatase (ALP), beta-galactosidase (β-GAL), and horseradish peroxidase (HRP). A chromogenic substrate is employed, and the color intensity of the solution is correlated with the analyte concentration. The enzyme–substrate reaction is stopped by adding a solution such as hydrochloric acid, sodium carbonate, sulfuric acid, or sodium azide, and the color signal is measured using a microtiter plate reader. ELISA offers advantages over instrumental analysis, including simplicity, high specificity, sensitivity, minimal sample preparation, and cost-effectiveness. Scholars (Ravi & Venkatesh, 2017) created an ic-ELISA for riboflavin detection in food and drugs using a D-ribitol antibody, achieving an LOD of 0.02 ng/mL and recovery rates between 90 % and 108 %. Researchers (T. Zhang et al., 2012) established an ic-ELISA for VB_9_ detection in vitamin-fortified foods, with LODs of 3.0 ng/mL in buffer, 3.52 ng/mL in energy drinks, 11.91 ng/mL in milk, and 16.50 ng/mL in milk powder, and recovery rates ranging from 88.3 % to 108.9 %. Some scholars (Kong et al., 2017) reported an ic-ELISA using a monoclonal antibody for VB_12_ detection in vitamin tablets, energy drinks, and infant milk powder, with an LOD of 0.065 ng/mL, an IC_50_ of 0.43 ng/mL, and recovery rates between 81 % and 122 %.

In summary, immunoassays are versatile and sensitive tools for detecting vitamins in various samples. Antibody-based immunoassays offer high specificity and accuracy, while the ongoing development of new haptens, antibodies, and immunoassay formats continues to enhance their application in food safety and quality control.

#### 3.5.4 Chemiluminescence immunoassay

The sensitivity of an immunoassay depends on the antibody sensitivity and the signal amplification system. CLIA combines immune responses with a chemiluminescence system, where a chemical probe labels the antibody, and the resulting light signal produced by a chemical reaction is used to determine the analyte concentration (Xiao & Xu, 2020). CLIA is highly sensitive, selective, and offers a broad dynamic range, ease of operation, and rapid results, making it popular in fields such as life sciences, clinical diagnostics, environmental testing, pharmaceutical analysis, and food safety. Innovations such as the use of AuNPs, MNMs, and QDs, along with technologies such as immunochromatography and microfluidic chips, are driving CLIA toward increased sensitivity, specificity, and throughput (Xiao & Xu, 2020). For example, scholars (X. Chen et al., 2019) developed a CLIA for detecting VB_9_ in human serum through a competitive reaction, achieving an LOD of 0.44 ng/mL, a recovery rate of 92.1–103.5 %, and low cross-reactivity with aminopterin, methylene acid, and methotrexate. Another CLIA developed by researchers (X. Chen et al., 2019) for detecting VB_12_ in human serum demonstrated an LOD of 41 pg/mL and a recovery rate of 90.7–107.4 %, showing excellent sensitivity and specificity compared with the Beckman Coulter system. Scholars (Hampel et al., 2014) reported a competitive CLIA for VB_12_ detection in human milk, validating the linearity, accuracy, matrix effects, precision, and robustness of the assay, with an LOD of 24 pM. Additionally, researchers (Han, Qiu, et al., 2020) designed a CLIA for 25-hydroxyvitamin D detection in human serum in which streptavidin-labeled magnetic particles were used to separate the signal-producing complex. The signal intensity was inversely related to the concentration of 25-hydroxyvitamin D, with an LOD of 1.43 ng/mL and a recovery rate of 93.22–107.99 %; these results were in agreement with similar CLIA methods reported by Roche and DiaSorin.

#### 3.5.5 Lateral flow immunoassay

LFIA, also known as a rapid diagnostic test (RDT) or immunochromatographic test strip (ICST), is a rapid assay that integrates immunochemical reactions with chromatographic techniques. In LFIA, molecular recognition elements, such as antigens or antibodies, are immobilized on a paper-based material with a porous structure that enables the filtration and migration of specific components through capillary action from complex sample matrices. When the target analyte in solution binds to an immunoreagent labeled with a signal marker (such as AuNPs, MNPs, CNPs, colored latexes, QDs, UCNPs, fluorescent dyes, and photoluminescent markers), the labeled immunoreagent undergoes aggregation, producing a detectable signal. This signal can be observed visually or measured instrumentally, allowing for qualitative or semiquantitative analysis. LFIA can detect targets in complex matrices within 15 min, making it an effective and versatile tool for resource-limited settings, such as field applications and developing regions where advanced instrumentation may be inaccessible. The global LFIA market reached $8.49 billion in 2022, and it is projected to grow at a compound annual growth rate (CAGR) of 4.7 %, reaching $13.38 billion by 2030 (Di Nardo et al., 2021).

LFIA has rapidly expanded into various fields, including veterinary medicine, food safety, environmental analysis, and clinical diagnostics (Mirica et al., 2022). The key advantages of LFIA, such as simplicity, speed, cost-effectiveness, and the ability to function without expensive equipment, make it widely applicable. Researchers (Z. Lu et al., 2017) created a sandwich LFIA diagnostic test combined with a mobile platform that integrates multiple fluorescent markers for the simultaneous detection of iron, VA, and inflammatory markers. The LFIA provided accurate quantification within 15 min with sensitivity rates of 88 %, 100 %, and 80 % for iron deficiency, VA deficiency, and inflammatory status, respectively, and specificity rates of 97 %, 100 %, and 97 %, respectively. Scholars (Liang et al., 2017) designed an LFIA for the rapid cost-effective detection of VB_9_ in maize seeds. The assay covered a detection range of 100–200 ng/mL for 5-formyltetrahydrofolate, 5-methyltetrahydrofolate, and their polyglutamate forms. Researchers (Novikova et al., 2020) developed an LFIA platform using AgInS/ZnS QDs as fluorescent markers for VB_9_ screening in fruit juice, achieving a visual cutoff of 3 μg/mL and an accuracy rate of 97.2 %. Scholars (Pan et al., 2021) synthesized osmium nanohydrangeas to generate color signals for VB_9_ detection, with a visual cutoff value of 100 ng/mL and an LOD of 0.01 ng/mL. Xu and colleagues constructed colloidal gold-based LFIA tests for the detection of VB_1_, VB_2_, VB_3_, VB_5_, VB_6_, VB_7_, VB_9_, and VB_12_. These results confirmed that the developed LFIA is effective for detecting these vitamins in infant milk powder, multivitamin supplements, and beverages.

As the number of detectable targets increases, a multichannel design is an ideal solution for LFIA. This approach allows for the simultaneous detection of multiple targets by incorporating multiple test channels into the test strip, enabling differentiation through spatial resolution. For example, scholars (J. Chen et al., 2023) developed a novel multiplex immunochromatographic strip utilizing fluorescent UiO-66-NH_2_@QDs for detecting five biotoxins, AFB_1_, FB_1_, DON, T-2, and ZEN, in grains and feeds. The LODs for these toxins were 0.04, 0.28, 0.25, 0.09, and 0.08 μg/kg, respectively. The recovery rates ranged from 82.83 % to 117.44 %, with CVs between 2.88 % and 11.80 %. Similarly, researchers (Lei et al., 2023) developed a multiplex AuNP-based LFIA for detecting residues of five antibiotic types (tetracyclines, β-lactam antibiotics, sulfonamides, quinolones, and phenylpropanols). The detection ranges for these antibiotics were 2.33–38.4 μg/kg, 0.688–17.1 μg/kg, 1.4–48.1 μg/kg, 1.45–32.9 μg/kg, and 0.537–9.06 μg/kg, respectively, with recovery rates between 87.5 % and 115.2 % and CV values below 9.5 %.

Vitamins, particularly FSVs, present significant challenges for multiplex assays because of their highly diverse chemical structures and numerous homologs. The development of multiplex assays capable of detecting multiple analytes in parallel within a single sample offers great potential for the qualitative screening and quantitative analysis of vitamins. In addition, single-signal output LFIAs can be affected by experimental variables, matrix interferences, environmental factors, and batch-to-batch inconsistencies. To overcome these limitations, a multimodal LFIA integrates multiple types of signal labels, which allows for multiple signal outputs from a single assay and avoids the inherent weaknesses of single-modal LFIAs. Advances in nanotechnology have facilitated the development of various multifunctional signal labels. For example, researchers (Z. Wang et al., 2024) synthesized multifunctional “dandelion-like” gold@platinum nanoparticles that offer fluorescence, colorimetric, catalytic, and photothermal signals. The resulting multimodal LFIA can output multiple detection signals, enhancing the practicality, flexibility, and accuracy of the assay. This technology holds promising potential as a point-of-care detection platform for various applications.

#### 3.5.6 Immunosensors

Immunosensors based on antibody–antigen-specific recognition are a distinct class of biosensors capable of accurate vitamin detection. Immunosensors can be categorized into unlabeled and labeled sensors on the basis of their analyte detection principles. Nonlabelled immunosensors directly measure the physicochemical changes resulting from antigen–antibody complex formation. These sensors are cost-effective, easy-to-operate, and typically used for detecting high-molecular-weight analytes at high concentrations. In contrast, labeled immunosensors, which utilize detectable tags that bind to antigens or antibodies, are suitable for ultrasensitive assays at low concentrations (Kausaite-Minkstimiene et al., 2024; H. Kumar et al., 2024).

Recent developments in immunosensors have enabled the detection of 25-OH VD_3_. For example, researchers (Sharma, Gupta, et al., 2024) modified a screen-printed paper electrode with GO to enhance the electrochemical signal, followed by functionalization with a monoclonal antibody for 25-OH VD_3_. This immunosensor, integrated with a smartphone android, achieved a sensitivity of 0.36 mA/mm^2^/pg and an LOD of 30.93 pg/μL for 25-OH VD_3_. Similarly, scholars (Kaur et al., 2023) synthesized a meso-microporous silica–metal organic framework (MSS-Z8) and further modified it with microcubic gold (MC–Au) to create an MC–Au/MSS-Z8 platform. The 25-OH VD_3_ antibody was adsorbed onto the platform, resulting in a broad linear detection range of 0.01–10^6^ pg/mL and an LOD of 0.23 pg/mL. In another approach (Anusha et al., 2023), scholars used a graphitic carbon nitride–β-cyclodextrin composite for electrochemical immunosensing, achieving a linear range of 0.00625–1.25 μM and an LOD of 0.0061 μM. Another innovative probe uses graphene nanoribbons (GNRs) and ferrocene–carbaldehyde (Fc-CHO)-conjugated antibodies. Additionally, an immunosensor incorporating cysteamine-functionalized core–shell magnetic nanoparticles (Au@MNPs) on a graphite screen-printed electrode demonstrated a linear range of 7.4–70 ng/mL and an LOD of 2.4 ng/mL (Polli et al., 2023). A voltammetric immunosensor based on molybdenum sulfide (MoS_2_) and AuNPs was developed, offering a linear range of 1 pg/mL–100 ng/mL and an LOD of 0.38 pg/mL (A. Kaur et al., 2021). Researchers (Chauhan et al., 2021b) created a BSA/anti-25VD₃/nCeO₂/CC nanobioplatform for 25-OH VD_3_ detection, achieving a broad linear range of 1–200 ng/mL and a quick response time of 15 min with an LOD of 4.63 ng/mL. Antibodies are sensitive to environmental conditions such as ionic strength, pH, and temperature, which may affect their stability (Zhenzhong Zhang et al., 2024). The development of stable microenvironments or the use of nanobodies can help maintain antibody activity for extended periods. Table 3 presents a summary of the reported immunoassays for vitamin detection, detailing the assay formats, signal labels, targets, LODs, and sample types.

**Table 3:** Summary of reported immunoassays for vitamin detection.

Immunoassays	Labels	Vitamins	Samples	LOD	Ref
Ic-ELISA	HRP	VB_1_	Energy drink, Vitamin tablet, infant milk	4.51 ng/mL	(Zeng et al., 2020)
Ic-ELISA	HRP	VB_2_	Vitamin drink, milk powder	24.6 ng/g, 0.5 mg/kg	(*P.*Wang et al., 2013)
Ic-ELISA	HRP	VB_2_	Energy drink, Vitamin tablet	1.80 ng/mL	(Zeng et al., 2018)
Ic-ELISA	ALP	VB_2_	Foods, capsule	0.02 ng/mL	(Ravi & Venkatesh, 2017)
Ic-ELISA	HRP	VB_5_	B-complex Vitamin tablets, energy drink, infant milk powder	32.22 ng/mL	(Zeng et al., 2021)
Ic-ELISA	HRP	VB_6_	Energy drink, B-Vitamin complex tablet	13.11 ng/mL 29.70 ng/mL 39.46 ng/mL	(Zeng et al., 2022)
Ic-ELISA	HRP	VB_9_	Energy drink, milk, milk powder	3.52 ng/mL, 11.91 ng/mL, 16.50 ng/mL	(T. Zhang et al., 2012)
Ic-ELISA	HRP	VB_9_	Energy drink, milk	0.018 ng/mL	(Kong et al., 2016b)
Ic-ELISA	HRP	VB_12_	Vitamin tablets, energy drink, infant milk powder	0.065 ng/mL	(Kong et al., 2017)
dc-ELISA	ALP	VB_12_	Multivitamin injections, tablets, capsules, chocolates	10 ng/mL	(Kumar & Thakur, 2011)
CLIA	HRP	VB_9_	Human serum	0.44 ng/mL	(X. Chen et al., 2019)
CLIA	HRP	VB_12_	Human serum	41 pg/mL	(X. Chen et al., 2020)
CLIA	ALP	VB_12_	Human milk	24 pM	(Hampel et al., 2014)
CLIA	HRP	25-OH-VD	Human serum	1.43 ng/mL	(Han, Wei, et al., 2020)
LFIA	PE/Cy5、FITC、RPE	VA	Human serum	14.7 μg/mL	(Z. Lu et al., 2017)
LFIA	AuNPs	VB_9_	Maize	Not reported	(Liang et al., 2017)
LFIA	AIS/ZnS quantum dots	VB_9_	Juice	3 μg/mL	(Novikova et al., 2020)
LFIA	osmium nanohydrangeas	VB_9_	Milk powder	0.01 ng/mL	(Pan et al., 2021)
LFIA	AuNPs	VB_1_	Energy drinks, vitamin tablets, infant milk powder	5.33 ng/mL	(Zeng et al., 2020)
LFIA	AuNPs	VB_2_	Energy drink, compound vitamin B_2_ tablet	50 ng/mL	(Zeng et al., 2018)
LFIA	AuNPs	VB_3_	Infant formulas, compound vitamin B tablets	116.13 ng/mL 20.76 ng/mL	(Hu et al., 2024)
LFIA	AuNPs	VB_5_	B-complex Vitamin tablets, energy drink, infant milk powder	71.99 ng/mL, 115.80 ng/mL, 240.12 ng/mL	(Zeng et al., 2021)
LFIA	AuNPs	VB_6_	Energy drinks, B-vitamin complex tablet	14.10 ng/mL, 55.58 ng/mL 56.25 ng/mL	(Zeng et al., 2022)
LFIA	AuNPs	VB_9_	Energy drink, milk	0.5 ng/mL	(Kong et al., 2016b)
LFIA	AuNPs	VB_12_	Infant milk powder, energy drink samples, vitamin tablets	1 ng/mL	(Kong et al., 2017)
Immunosensor	nCeO_2_/CC	25-OH VD_3_	human serum	4.63 ng/mL	(Chauhan et al., 2021a)
Immunosensor Immunosensor	Au@MNPs Au-MoS_2_	25-OH VD_3_ 25-OH VD_3_	human serum human serum	2.4 ng/mL 0.38 pg/mL	(Polli et al., 2023) (A. Kaur et al., 2021)
Immunosensor Immunosensor	GCN-β-CD/ AuNPs OsNHs	25-OH VD_3_ VB_9_	human serum milk powder	0.01 ng/mL 100 ng/mL	(Anusha et al., 2022)
Immunosensor	MC-Au/MSS-Z8	25-OH VD_3_	human serum	0.23 pg/mL	(Amandeep Kaur et al., 2023)

### 3.6 Others

In addition to the detection techniques discussed earlier, this section is focused on a commonly used biosensor for vitamin detection: the aptamer sensor (aptasensor) are biosensors that generate an analytical signal from the binding interaction between a target molecule and an aptamer, which is a short, single-stranded DNA or RNA molecule with high affinity and specificity for its target (Borg et al., 2024). Aptamers possess compelling features owing to their synthetic nature, such as easy chemical modification, high specificity, long storage life, stability, low cost, and minimal batch variation (Azzouz et al., 2023). Aptasensors are gaining attention in the fields of diagnostics, environmental monitoring, and food safety, where they offer benefits such as low production costs, minimal batch-to-batch variations, long shelf lives, and well-controlled binding properties. Leveraging the addressability, rigidity, and biological activity characteristics of DNA, immobilized DNA on a sensor surface via physical adsorption, covalent bonding, or affinity interactions enhances the sensor performance by increasing the surface area for aptamer binding, increasing the sensitivity, and reducing the detection limits. For example, scholars (Jo et al., 2021) developed an aptasensor utilizing PEG-free gold nanorods (AuNRs) functionalized with aptamers for 25-OH VD_3_ detection, which displayed good linearity from 0.1 to 10^5^ ng/mL with an LOD of 0.1 ng/mL. Additionally, researchers (Kim et al., 2021) employed a bioinspired Ag nanovilli (AgNV)-based sandwich-type surface-enhanced Raman spectroscopy (SERS) aptasensor for 25-OH VD_3_ detection. The SERS aptasensor demonstrated high sensitivity, with an LOD of 0.001 ng/mL for 25-OH VD_3_. Scholars (Yin et al., 2022) designed an enzyme-free aptasensor using catalytic hairpin assembly reaction and DNA tetrahedron modification processes, establishing a linear range spanning from 0.1 to 1000 nM and an LOD of 0.026 nM.

## 4 Limitations and challenges

This article reviews recent advancements in vitamin analysis in various sample matrices. As essential micronutrients, vitamins are critical for health assessment and quality assurance. However, vitamin deficiency remains a persistent concern. In recent years, considerable efforts have been directed toward enhancing the sensitivity, accuracy, robustness, and validity of analytical methods for vitamin detection in diverse matrices. However, vitamin analysis continues to face challenges, particularly due to the inherent properties of vitamins and the complex compositions of sample matrices. For example, the intricate compositions of food, pharmaceutical, and clinical samples introduce various compounds that can interfere with accurate vitamin quantification. Addressing matrix interference requires analytical methods with improved selectivity and resistance to matrix effects to ensure robust and accurate results. The rapid advancement of nanomaterials has created new possibilities in spectroscopic and electrochemical analyses. Despite the establishment of numerous platforms for vitamin analysis, challenges remain in terms of stability, accuracy, and reproducibility in practical applications. Among these methods, LC–MS remains the most reliable and robust technique, often serving as a benchmark for verifying other detection methods. Recent innovations in high-resolution mass spectrometry and advanced chromatography have further extended LC–MS applications in vitamin detection. However, the time-intensive nature and high costs of LC–MS restrict its use for rapid, onsite applications, especially in resource-limited settings. While highly sensitive biosensors have been developed, their practical application in laboratory settings remains constrained by issues such as accuracy, precision, and traceability. Immunoassays that rely on specific antigen–antibody binding are a promising alternative for high-throughput vitamin analysis. However, the continuous optimization of antigen and antibody stability and signal amplification systems is necessary.

Despite the significant progress made in vitamin analyses in different sample matrices, several challenges remain:

(1) Extraction and purification procedures. Vitamins are chemically unstable and susceptible to degradation by light, pH changes, oxygen, heat, and other factors, in addition to binding with proteins, lipids, and other molecules. To minimize matrix interference, the development of novel materials, eco-friendly solvents, and efficient sample clean-up procedures is essential.
(2) Method integration. Existing detection methods each have limitations, making it necessary to integrate multiple detection techniques, such as optical analysis, electrochemical analysis, and immunosensor analysis, or to combine these techniques with chromatographic methods for the cross-validation of analyte measurements. The continuous optimization and integration of a wide range of instruments and mechanisms are crucial for achieving vitamin assays with high reliability, accuracy, reproducibility, precision, and sensitivity.
(3) Personalized customization. Given the distinct chemical structures of various vitamins, customizing detection platforms by leveraging new materials and tailored sensing mechanisms is essential. Such customization enables the development of portable, real-time, and high-throughput vitamin detection platforms that are suitable for specific vitamins.
(4) Commercial kits. Similar to microbial assay kits for water-soluble vitamins, immunoassays and commercial kits are promising options for vitamin analysis. The demand for rapid, convenient, and cost-effective vitamin assays, particularly in point-of-care settings, underscores the need for the further development of these kits.

## 5 Conclusions

In this review, detection methods for vitamins are comprehensively explored, emphasizing the importance of analytical techniques in enhancing detection accuracy and reliability. The development of miniaturized and portable devices has led to a shift toward onsite and point-of-care vitamin analysis, enabling real-time quality and safety monitoring. Concurrently, the rise of multimodal analytical approaches, which integrate certain fields, such as spectroscopy, chromatography, immunology, and materials science, marks a transformative phase in vitamin analysis. Additionally, combining artificial intelligence, big data analysis, and other advanced technologies is expected to drive the automation and intelligence of vitamin testing, enhancing both the efficiency and accuracy of detection. These advancements hold promise for ensuring human health and product quality through improved methodologies and a more comprehensive understanding of vitamin analysis. Addressing these challenges necessitates interdisciplinary collaboration and ongoing innovation to meet health and industry standards.

## CRediT authorship contribution statement

**Xiangrui Li:** Writing – original draft, Software, Methodology, Data curation, Conceptualization. **Huan Lv:** Writing – review & editing, Methodology, Investigation. **Wencan Luo:** Methodology, Investigation. **WenJia Yang:** Methodology, Investigation. **Linghong Kong:** Methodology, Investigation. **Qiujin Zhu:** Supervision, Resources, Project administration. **Lu Zeng:** Writing – review & editing, Supervision, Resources, Project administration, Funding acquisition.

## Declaration of competing interest

The authors declare that they have no known competing financial interests or personal relationships that could have appeared to influence the work reported in this paper.

## Data Availability

Data will be made available on request.
